# Phenotypic variation of root-system architecture under high P and low P conditions in potato (*Solanum tuberosum* L.)

**DOI:** 10.1186/s12870-023-04070-9

**Published:** 2023-02-01

**Authors:** Julian Kirchgesser, Mousumi Hazarika, Silvia Bachmann-Pfabe, Klaus J. Dehmer, Mareike Kavka, Ralf Uptmoor

**Affiliations:** 1 Department of Agronomy, University of Rostock, Justus-Von-Liebig-Weg 6, 18059 Rostock, Germany; 2 Leibniz Institute of Plant Genetics and Crop Plant Research, Groß Luesewitz Potato Collection, Parkweg 3, 18190 Sanitz, Germany; 3 Neubrandenburg University of Applied Science, Brodaer Str. 2, 17033 Neubrandenburg, Germany

**Keywords:** Root system, Phosphorus deficiency, Genetic resources, Rhizotrons

## Abstract

**Background:**

Phosphorus (P) is an essential macronutrient required for plant metabolism and growth. Its acquisition by plants depends on the availability of dissolved P in the rhizosphere and on the characteristics of P uptake mechanisms such as root-system architecture (RSA). Compared to other crops, potato (*Solanum tuberosum* L.) has a relatively poor P acquisition efficiency. This is mainly due to its shallow and sparsely branched root system, resulting in a rather limited exploitable soil volume. Information about potato genotypes with RSA traits suitable to improve adaptation to nutrient scarcity is quite rare. Aim of this study is to assess phenotypic variation of RSA in a potato diversity set and its reactions to P deficiency.

**Results:**

Only one out of 22 RSA-traits showed a significant increase under low-P conditions. This indicates an overall negative effect of P scarcity on potato root growth. Differences among genotypes, however, were statistically significant for 21 traits, revealing a high variability in potato RSA. Using a principal component analysis (PCA), we were able to classify genotypes into three groups with regard to their root-system size. Genotypes with both small and large root systems reacted to low-P conditions by in- or decreasing their relative root-system size to medium, whereas genotypes with an intermediate root system size showed little to no changes.

**Conclusions:**

We observed a huge variation in both the potato root system itself and its adaptation to P deficiency. This may enable the selection of potato genotypes with an improved root-zone exploitation. Eventually, these could be utilized to develop new cultivars adapted to low-P environments with better resource-use efficiencies.

## Background

Phosphorus (P) is a key component of many important biomolecules such as DNA, ATP and phospholipids, making it essential for plant growth and metabolism [1–3]. In agriculture, biomass removal and erosion lead to constant P losses and make P-fertilizer application crucial for sustaining high yields [4, 5]. In this context, rock-phosphate based mineral fertilizers represent the most important commercially used P source. The natural P reserves, however, are limited, require considerable technical effort and are often located in politically fragile countries [6, 7]. Another important aspect of P usage is its impact on the environment, since excessive fertilization can lead to severe problems such as eutrophication of neighboring water bodies and harmful algae bloom [8]. Thus, careful P handling and an efficient use of soil-available P should be targeted.

In plants, P uptake from the soil occurs via inorganic compounds such as orthophosphate in the form of HPO_4-_ and H_2_PO_4-_ [9, 10]. However, P immobilizes rapidly in most soils and forms stable complexes with clay minerals and other elements such as calcium, magnesium, aluminum and iron cations [11]. Metabolization of P sources by microorganisms can also affect P availability [12]. High P application in combination with its low mobility leads to topsoil accumulation of phosphate [13].

During evolution, several strategies of adaptation to P deficiency have been developed. This includes changes in root morphology and architecture [14, 15]. Root-morphology based mechanisms to enhance P acquisition are mainly focused on increasing soil exploration [16, 17]. The spatial organization of primary roots as well as root- and stem-derived branches (lateral roots) and total root length play a crucial role in the adaptation to unfavorable soil conditions [18]. Since root growth and development are highly affected not only by environmental factors but also by genetic components, the characterization of RSA may enable breeders to select plants with improved P-uptake efficiency [19]. Most studies on crop root systems addressed the major cereal crops wheat and maize and were related to drought tolerance [20–23]. Parra-Londono et al. characterized the reactions of sorghum RSAs on P deficiency using 200 genotypes, while most other studies comprised a smaller number of genotypes. A high variability in both sorghum RSA and adaptation to P deficiency was observed [19]. Potato root morphology and root-system adaptation to drought stress was evaluated in both pot and field experiments [24–26]. Effects of P deficiency on root growth and development have been described in only a few studies, which used a small number of genotypes [27, 28]. Many small-plant approaches used filter paper, agar or (semi-) hydroponics to evaluate root systems of crops [21, 22]. Rhizotrons have the advantage that soil based growing media can be used. A main disadvantage is the time-consuming root washing, which is required for any further analysis like dry weight measurements. Root growth can be monitored in glass-walled rhizotrons [22], however, roots are generally not growing perfectly along the glass or acrylic-glass walls of rhizotrons. Hylander described a method to analyze soil-root interactions and monitor 2D-root growth in rhizotrons by placing nylon meshes between the soil and rhizotron walls [29]. In this way improved rhizotrons were used for high-throughput phenotyping of wheat and sorghum root systems [19, 23] but—according to our knowledge—not for dicots so far.

The potato (*Solanum tuberosum*, L) is the most important non-cereal crop in the world in terms of agricultural output [30]. Besides being a major staple food, it is an important starch crop for the industry as well. Potato starch features top-level quality since its pastes have a good clarity due to small amounts of lipids and proteins as well as a good solubility and viscosity compared to other starch sources [31]. An optimal P supply not only affects the general growth of potato plants but also improves the quality of both tubers and starch. Research studies showed that enhancing the potato-starch phosphate content leads to increases in swelling power, peak viscosity and breakdown viscosity as well as to significant but small increases in onset and peak temperatures of gelatinization [32].

Compared to other crops such as rice, maize or pigeon pea, the P-uptake efficiency of potatoes is relatively poor. This is mainly due to their shallow and sparsely branched root system, resulting in a rather limited soil volume exploitable by the plant [28, 33]. Studies on several potato genotypes give hint that a considerable genetic variation exists in both root architecture and adaptation of the root system to P deficiency [30]. We hypothesize that a certain variation in RSA enables the selection of genotypes with improved P acquisition efficiency. The main objectives of the present study are to characterize RSA of a potato diversity set and to assess the genetic variation of both RSA itself and adaptations of the potato RSA to P deficiency in order to identify genotypes with advantageous root systems under non-stress and low-P conditions.

## Material and methods

### Experiments to assess traits related to RSA

A potato diversity set comprising 200 genotypes was used in the present study. The plant material included 195 entries of the crop species *S. tuberosum* and five from other wild and cultivated South American *Solanum* species. It was provided by the Gene Bank of the Leibnitz Institute of Plant Genetics and Crop Plant Research (IPK). The plant material comprised mainly (formerly) registered varieties and was identified as *S. tuberosum* by the respective federal variety offices or upon entry into the IPK Genebank by the responsible curator of the IPK Gross Luesewitz Potato Collections (GLKS). Due to the lack of substantial morphological differences, no herbarium vouchers of cultivated potatoes are deposited at the GLKS herbarium. However, DNA samples of all entries of this study are deposited at IPK and are available upon request. Herbarium vouchers of material other than *S. tuberosum* have been taken upon their first cultivation at IPK and are deposited at the GLKS herbarium and can be studied upon request. The *S. tuberosum* material used in this study was not collected but obtained either from the IPK Genebank, which has received the respective material before the entering into force of the Convention of Biological Diversity or under the conditions of the Standard Material Transfer Agreement of the International Treaty on Plant Genetic Resources for Food and Agriculture. Thus, all legal regulations concerning plant genetic resources are met. Alternatively, several entries were donated directly from the breeders of the material. All methods were done in accordance with national and international guidelines for plant experiments.

**Fig. 1:**
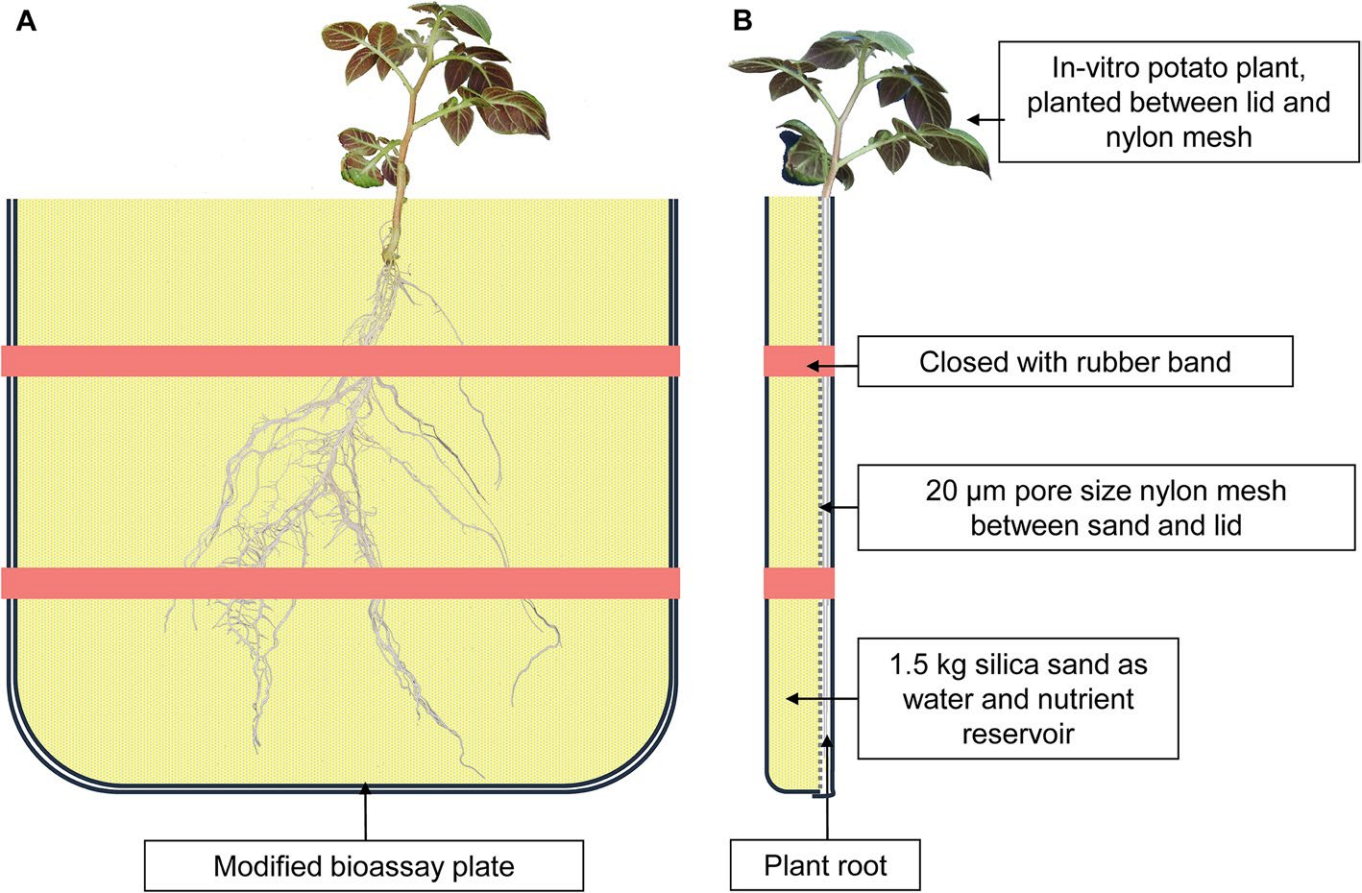
Schematic drawing of the front (**A**) and side view (**B**) of the 24 × 22 cm rhizotrons used for root phenotyping

Most *S. tuberous* genotypes are from Europe while some originate from Asia, Africa and the Americas. The diversity set comprises 68 starch, 60 fresh and 13 processing potato genotypes. Another 24 genotypes are suitable for universal uses while 37 have no known specialty. The diversity set includes 17 modern cultivars provided by breeders. Genotypes used in the RSA experiment were pre-cultivated as *in-vitro* plants at IPK’s Gross Luesewitz Potato Collections (GLKS). Experiments were carried out in May/June and June/July 2020 and plants were cultivated for ten days at a day/night air temperatures of 22/18 °C at the University of Rostock. The used mini-rhizotrons were built from polystyrene square bioassay plates (24 × 24 cm, Thermo Scientific™ Nunc™) and nylon meshes with 20 μm pore size (Klein & Wieler, Königswinter, Germany). The top of each bioassay plate was cut with a bandsaw (Fig. 1). A nylon mesh was attached between the bottom plate and the lid of the bioassay plate as described before [19, 29]. Each mini-rhizotron had been filled with 1.5 kg of silica sand (granule sizes 0.4—0.8; 0.71—1.25; 1.2—2.5 mm). Rubber bands were used to keep parts together. A modified Hoagland solution (pH 5,8) containing 5 mM KNO_3_, 5 mM Ca(NO_3_)_2_ × 4H_2_O, 2 mM MgSO_4_ × 7H_2_O, 0.1 mM FeNa-EDTA × 3 H_2_O, 25.07 μM H_3_BO_3_, 2.01 μM MnSO _4_ × 2H_2_O, 2.02 μM ZnSO_4_ × 7H 2O, 0.52 μM CuSO_4_ × 5H_2_O, 0.50 μM Na_2_MoO_4_ × 2H_2_O and 50.3 μM KCl was used as fertilizer. P concentrations were adjusted to 0.5 mM (HP) and 0.1 mM (LP) using KH_2_PO_4_. Potassium was added as KCl to both solutions to a total concentration of 1 mM K. For initial watering, rhizotrons were treated with 100 mL of the HP or LP solutions, respectively. For planting the rhizotrons were opened and *in-vitro* plants were placed between the lid and the nylon mesh so that roots were completely trapped between both (Fig. 1). The nylon mesh prevented roots from penetrating into the sand compartment, while water, nutrients, and root exudates were able to pass through. Mini-rhizotrons were stacked at an angle of ~ 70° into plastic boxes, which were covered with transparent plastic bags during the first 3 days after planting to avoid transpiration losses. The 200 genotypes were randomly arranged in HP- or LP-only boxes. The eleven plants per box were also randomly arranged. The experiment was conducted twice to obtain two replications per P treatment. Using a multichannel pipette, 7 mL of the same modified Hoagland solutions used for initial fertilization were applied every day from the start of the experiment. Roots and shoots were harvested 10 days after planting, when most roots were reaching the bottom of the rhizotrons.

### Root-image acquisition and analysis

**Fig. 2:**
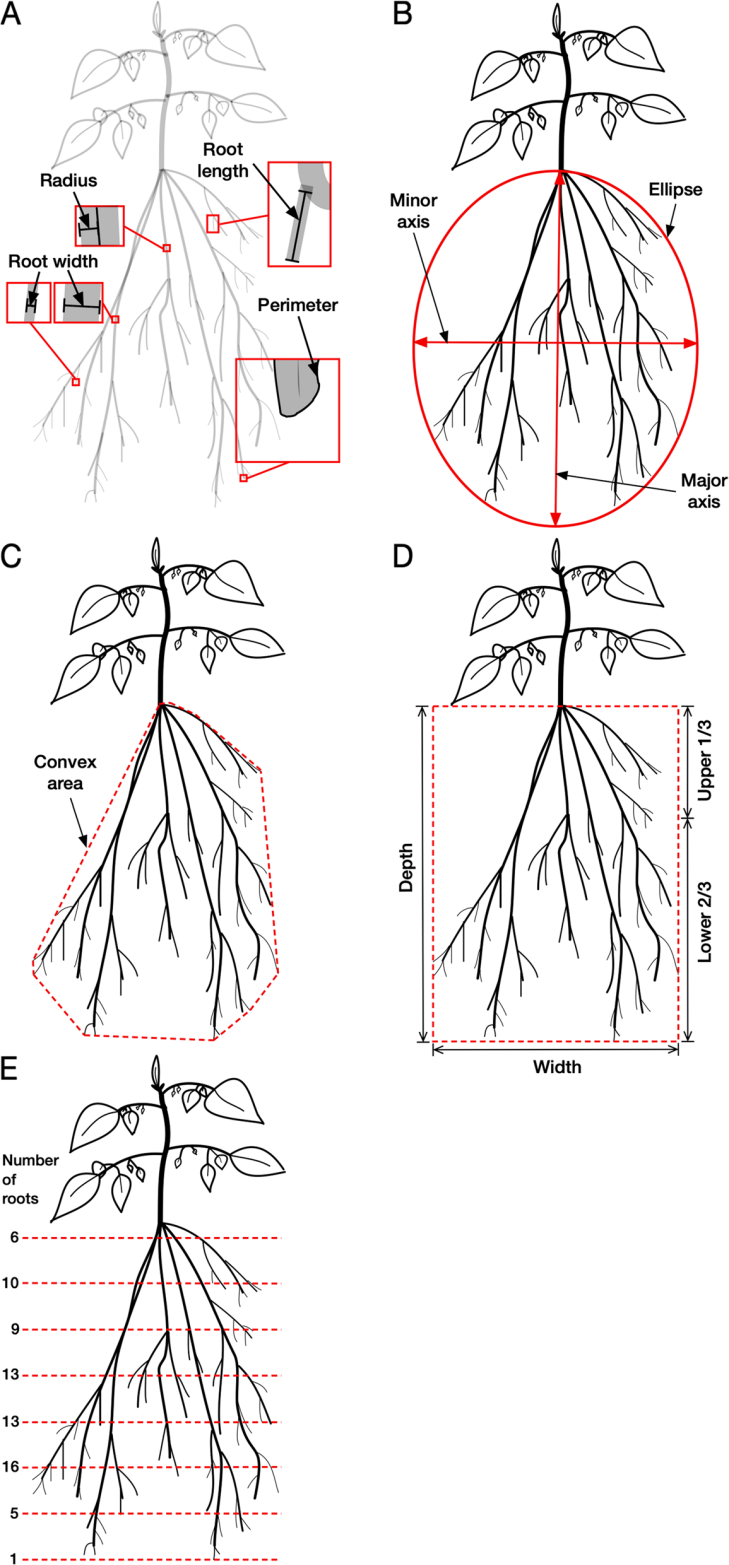
Radius for calculating network volume and surface area, root length, width and the perimeter encompassing the 2D figure of a root (**A**). Major and minor axis of the best fitting ellipse enclosing the root network (**B**). Convex area encompassing the root network (**C**). Rooting depth and width (**D**). Number of roots crossing horizontal lines (**E**)

At harvest, shoots were separated from roots and root systems were carefully transferred to a scanner (CanoScan LiDE 120, Canon, Tokyo, Japan) without disturbing the root architecture. Images were acquired with an image resolution set to 300 dpi. A black background was used to maximize contrast. Image processing was carried out with GiA Roots [34], which is a free software package designed for root-system phenotyping. Nineteen root parameters describing size, extent, shape and distribution of the root network were analyzed. A detailed description of the traits is given in Table 1. Schematic descriptions of all traits can be found within the GiA Roots software in the section “Features & Algorithms” [35]. The parameters used for calculating root traits by GiA Roots are shown in Fig. 2. Furthermore, root dry weight, shoot dry weight, and root-to-shoot ratio were determined.

**Table 1:** Abbreviations, units, root trait categories and descriptions of root traits analyzed* with GiA Roots* [34, 36]

Trait	Abbreviation	Category	Description
Shoot dry weight	SDW	Weight	Dry weight of the shoot after drying at 60 °C
Root dry weight	RDW	Weight	Dry weight of the root after drying at 60 °C
Shoot-to-root ratio	SRR	Weight	Ratio of the shoot-to-root weight after drying at 60 °C
Average root width	ARW [cm]	Size	Mean value of the root width estimation computed for all pixels of the medial axis of the entire root system (Fig. 2A)
Network area	NeA [cm^2^]	Size	Number of network pixels in the image
Network length	NeL [cm]	Size	Total number of pixels in the network skeleton (total root length, Fig. 2A)
Network perimeter	NeP [cm]	Size	Total number of pixels connected to a background pixel (perimeter of the root system, Fig. 2A)
Network surface area	NeSA [cm^2^]	Size	Sum of the local surface area at each pixel of the network skeleton, as approximated by a tubular shape whose radius (Fig. 2A) is estimated from the image
Network volume	NeV [cm^3^]	Size	Sum of the local volume at each pixel of the network skeleton, as approximated by a tubular shape whose radius (Fig. 2A) is estimated from the image
Major ellipse axis	MaEA [cm]	Extent	Length of the major axis of the best fitting ellipse enclosing the network (Fig. 2B)
Minor ellipse axis	MiEA [cm]	Extent	Length of the minor axis of the best fitting ellipse enclosing the network (Fig. 2B)
Network convex area	NeCA [cm^2^]	Extent	Area of the convex hull that encompasses the root system (Fig. 2C)
Network depth	NeD [cm]	Extent	Number of pixels in the vertical direction from the uppermost network pixel to the lowermost network pixel (Fig. 2D)
Network width	NeW [cm]	Extent	Number of pixels in the horizontal direction from the leftmost network pixel to the rightmost network pixel (Fig. 2D)
Maximum number of roots	MaNR	Ditribution	After sorting the number of roots crossing a horizontal line from smallest to largest, the maximum is considered to be the 84th percentile value (Fig. 2E)
Median number of roots	MeNR	Distribution	Result of a vertical line sweep, in which the number of roots that crossed a horizontal line was estimated and then the median of all values for the extent of the horizontal network was calculated
Network bushiness	NeB	Distribution	Ratio of maximum to median number of roots (Fig. 2E)
Network length distribution	NeLD	Distribution	Fraction of the network pixel found in the lower two-thirds of the network (Fig. 2 D)
Network solidity	NeS [cm^2^ cm^−2^]	Distribution	Total network area divided by the convex area (Fig. 2C)
Specific root length	SRL [cm cm^−3^]	Distribution	Total root length divided by the network volume (Fig. 2A)
Ellipse axes ratio	EAR [cm cm^−1^]	Shape	Ratio of the minor to the major axis of best-fitting ellipse (Fig. 2B)
Network width to depth ratio	NeWDR	Shape	Value of the network width divided by the value of the network depth (Fig. 2D)

### Data analysis

Two factorial analysis of variance, principal component (PCA) and cluster analysis were carried out using R version 3.6.3. Broad sense heritability was calculated as h^2^ = σ_g_ / (σ_g_ + σ_ge_ / 2 + σ_gr_ / 2 + σ_ger_ / 4), where σ_g_ is the genotype variance and σ_ge_, σ_gr_ and σ_ger_ are the genotype × environment, genotype × replication and genotype × environment × replication interaction variances. Since the second replication was conducted two months after the first one, the replications were not used for estimating error variance but considered like an „year “ effect in conventional h^2^ equations. Therefore the term for the error variance is missing in our equation. As a result, heritabilities may have been overestimated and should be used only to compare values of the present study. A correlation matrix was drawn with R using the packages “reshape2”, “ggplot2” and “Hmisc”. RSA classification of potato genotypes was carried out following the procedure described by Bodner et al. [37], which is based on principal components and computed with the R package “FactoMineR”. Cluster analysis was done using the hierarchical agglomerative method implemented in the R package “hclust”. Prior to PCA and cluster analysis, root trait means obtained for each genotype in each growth condition were standardized by using z-scores. For better comparison, a tanglegram consisting of both high phosphorus (HP) and low phosphorus (LP) dendrograms with auxiliary lines to connect genotypes in both dendrograms was created using the R package “dendextend” [38].

## Results

### P scarcity results in smaller root systems

Among the 200 potato genotypes differences were statistically significant for 21 out of 22 RSA traits (Table 2). ANOVA revealed that 15 traits were significantly different between HP and LP environments. The genotype × treatment interaction was significant for the network width to depth ratio (NeWDR). Heritability was on a similar level for most of the analyzed traits. Extreme low heritabilities were found for network bushiness (NeB) and ellipse axes ratio (EAR).

**Table 2:** Means, standard deviations (s.d.), heritabilities (h^2^) and analysis of variance (ANOVA) results for environment (E), genotype (G) and E × G*-*interaction effects

Trait^a^	Category	HP		LP		ANOVA^b^			h^2^
		Mean	s.d	Mean	s.d	E	G	E × G	
SDW [g]	Weight	0.065	0.021	0.062	0.020	**	***	NS	0.75
RDW [g]	Weight	0.026	0.011	0.024	0.010	***	***	NS	0.70
SRR	Weight	0.396	0.102	0.388	105	NS	***	NS	0.49
ARW [cm]	Size	0.047	0.005	0.047	0.006	NS	***	NS	0.42
NeA [cm^2^]	Size	13.600	5.764	12.445	5.304	***	***	NS	0.67
NeL	Size	360.21	165.210	329.47	155.63	***	***	NS	0,65
NeP [cm]	Size	669.35	311.971	610.56	292.16	***	***	NS	0.65
NeSA [cm^2^]	Size	51.975	22.030	47.567	20.333	***	***	NS	0.66
NeV [cm^3^]	Size	0.764	0.308	0.704	0.281	***	***	NS	0.66
MaEA [cm]	Extent	19.166	3.073	19.467	2.949	NS	***	NS	0.62
MiEA [cm]	Extent	8.301	3.172	7.783	2.883	**	***	NS	0.31
NeCA [cm^2^]	Extent	163.12	71.746	154.08	68.901	*	***	NS	0.54
NeD [cm]	Extent	20.516	3.038	20.657	3.045	NS	***	NS	0.67
NeW [cm]	Extent	10.365	4.047	9.821	3.658	*	***	NS	0.43
MaNR	Distribution	13.583	4.464	12.432	4.209	***	***	NS	0.51
MeNR	Ditribution	7.869	3.149	7.096	2.905	***	***	NS	0.53
NeB	Distribution	1.817	0.468	1.838	0.424	NS	N.S	NS	0.06
NeLD	Distribution	0.541	0.258	0.590	0.285	**	***	NS	0.28
NeS [cm^2^ cm^−2^]	Distribution	0.088	0.024	0.086	0.023	NS	***	NS	0.45
SRL [cm cm^−3^]	Distribution	466.13	95.740	461.53	96.642	NS	***	NS	0.44
EAR [cm cm^−1^]	Shape	0.433	0.154	0.401	0.138	***	*	NS	0.00
NeWDR	Shape	0.499	0.173	0.470	0.150	**	***	*	0.18

^a^ Trait abbreviations are explained in Table 1

^b^ *P < 0.05; **P<0.01; ***P<0.001; NS, not significant

Negative impact of P deficiency was ascertained for most root traits describing weight, size and distribution, indicating an overall negative effect of P deficiency on root growth. The extent of the root system was smaller at low P as well. The root network had a significantly decreased width (NeW), showing that plants did not react on the decreased P availability with increased exploitation of the soil volume in the present study. Soil volume was, of course, limited by the rhizotron size. At LP, the network lengths distribution (NeLD), describing the fraction of the root network found in its lower two-thirds, increased.

**Fig. 3:**
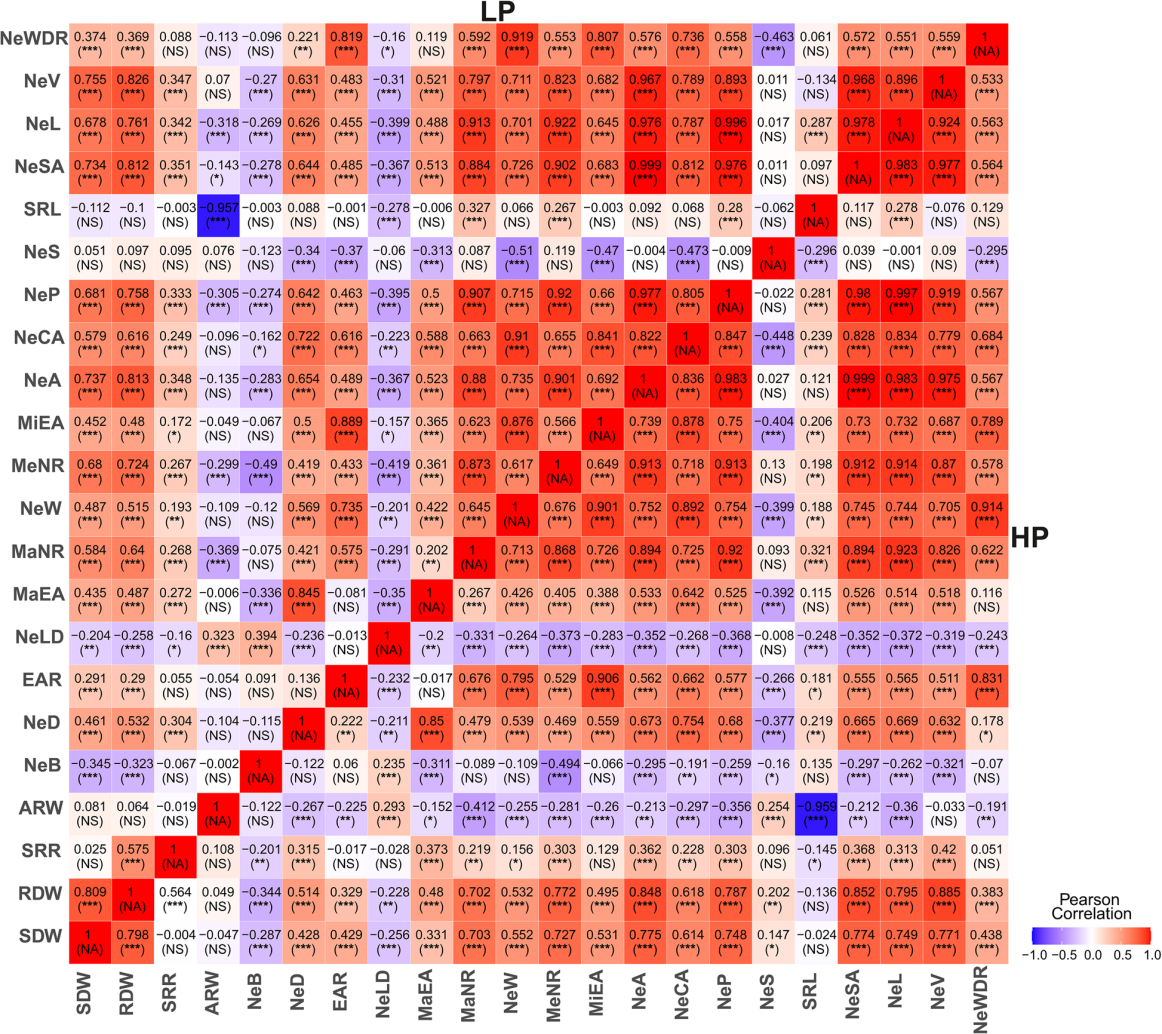
Heat map showing Person’s correlation coefficients among z-score standardized root traits assessed at high (HP) and low P (LP). *P < 0.05; **P < 0.01; ***P < 0.001; NS, not significant; NA, not available

With the exception of the average root width (ARW), both HP and LP data showed significant positive correlations for traits belonging the categories weight, size, extent and distribution (Fig. 3). Pearson’s correlation coefficients were particularly high when comparing maximum number of roots (MaNR), medium number of roots (MeNR), minor ellipse axis (MeNR), network perimeter (NeP), network surface area (NeSA), network width (NeW), network area (NeA), network convex area (NeCA), network length (NeL), and network volume (NeV) with each other. Average root width (ARW), network length distribution (NeLD), network bushiness (NeB), and network solidity (NeS) were negatively correlated with almost all other RSA traits.

### Classification of root systems by cluster analysis and PCA revealed three rooting types

**Fig. 4:**
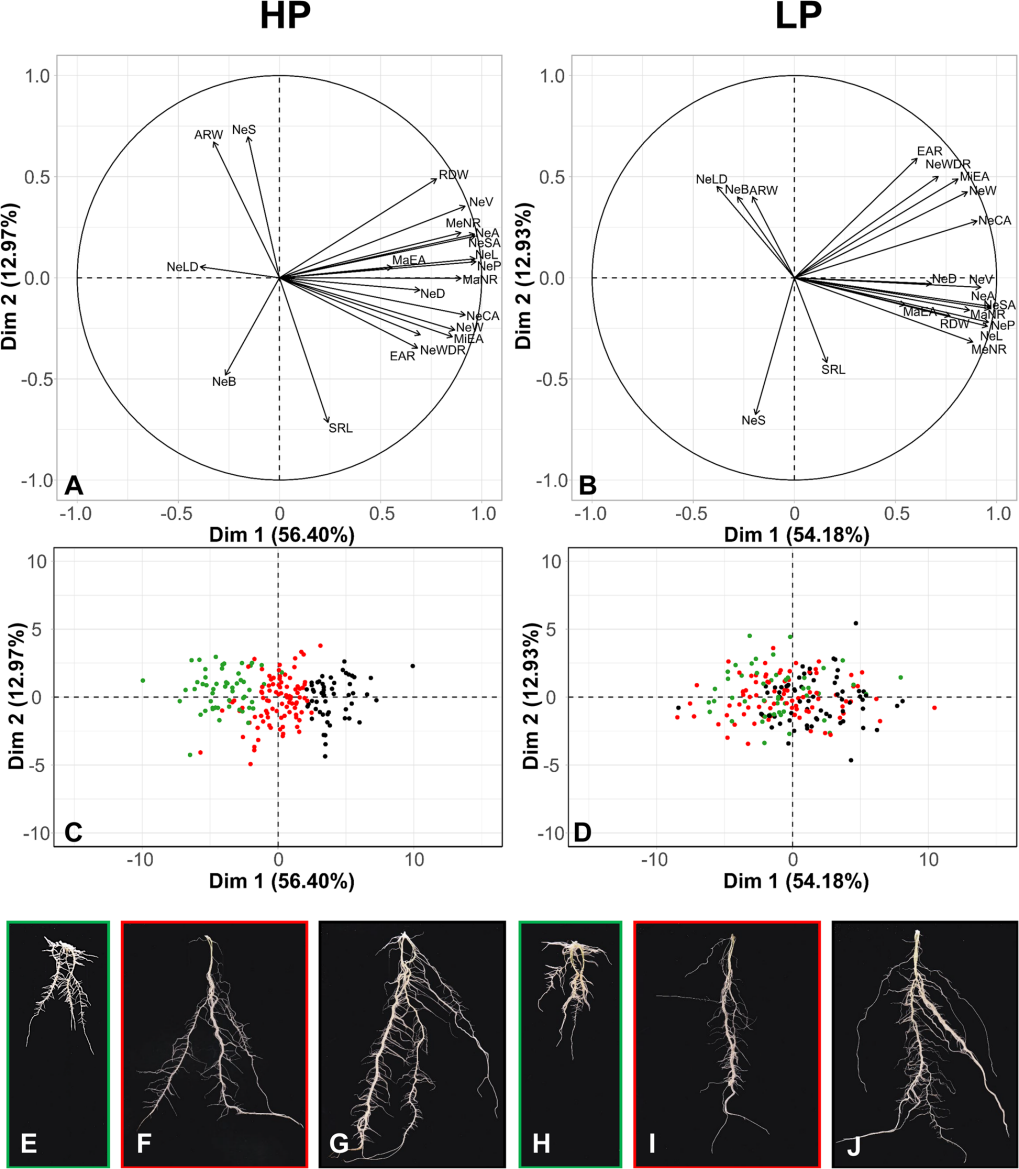
Biplots generated from PCA with the percentage of variance explained by the first two principal components (Dim 1, Dim 2) for root traits in high P (HP) and in low P (LP). Directional vectors represent traits and dots represent potato genotypes. Dot colors display the rooting types “small” (green), ”medium” (red) and “large” (green) as defined by cluster analysis at HP. Typical root systems belonging to the clusters “small“ (E, H), “medium“ (F, I) and “large“ (G, J) according to cluster analysis. E-G are from the HP and H-J from the LP treatment. E and H, F and I, and G and J are the same genotypes

**Fig. 5:**
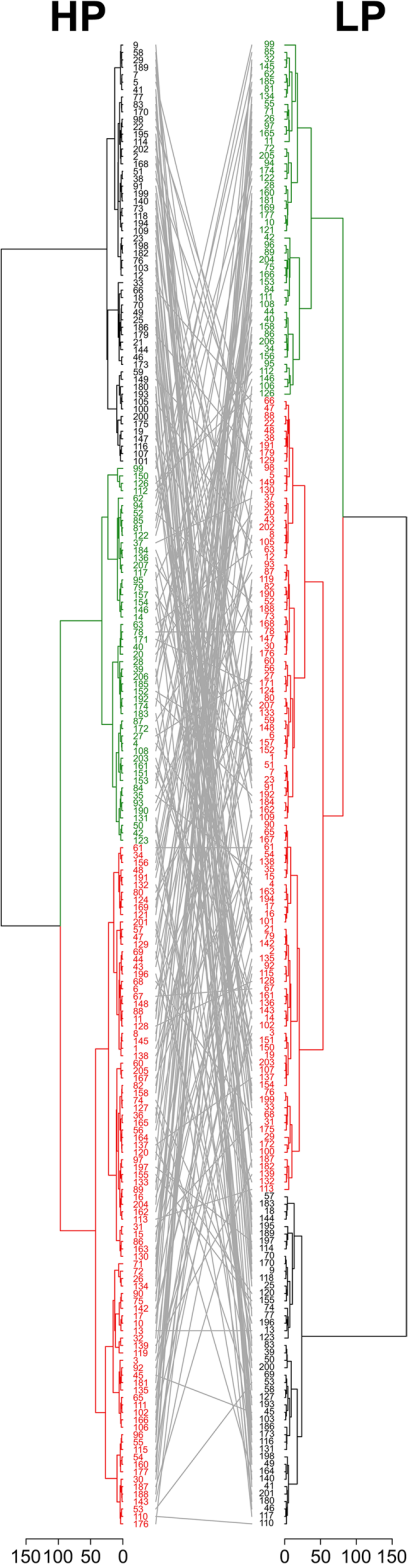
Tanglegram consisting of both high phosphorus (HP) and low phosphorus (LP) dendrograms with auxiliary lines to connect genotypes in both trees. Dendrograms display clusters with “small” (green), “medium” (red) and “large” (black) rooting types in HP and LP, respectively

Root-trait based PCA (Fig. 4) and cluster analysis (Fig. 5) classified potato genotypes into three distinct groups, which corresponded to their root-system size. Under both HP and LP conditions, the first principal component (Dim1) accounted for more than 50% of RSA variation (Fig. 4A-D).

Vectors describing network weight, size and extent were strongly aligned in the direction of the positive Dim1, indicating high variation within the diversity set for these traits. Traits describing root distribution were located in the direction of the second principal component (Fig. 4A and B).

Root systems classified at HP as “small” cluster in the negative direction of the first principal component, whereas the “medium” cluster is in the center and the “large” cluster is oriented towards the positive direction (Fig. 4C). In the LP biplot, genotypes are colored as defined by cluster analysis in HP and tend to cluster more in the center of the PCA, indicting a trend of developing medium-sized RSAs under those conditions (Fig. 4D).

More detailed information about RSA classification is presented in the tanglegram (Fig. 5). It consists of both HP and LP dendrograms with auxiliary lines connecting the same genotypes in both trees. Results show some consistency concerning RSA. In total, 46.5% of all genotypes show the same RSA classification at HP and LP and another 50.5% vary by one category (e.g. from “medium” to “small” or “large”). 37.3% of all genotypes categorized as “small” in the HP treatment remained small in LP, whereas the majority (50.9%) became “medium” and 11.8% were considered as “large” in LP. Among genotypes with “medium” sized RSA in HP, the majority (53.3%) remained “medium” in the LP condition. 31.5% were getting smaller and 15,2% developed “large” RSAs. From the “large” RSA category in HP, 43.9% remained in the “large” category at LP, whereas the majority of 56.1% became “medium”. A change from “large” to “small” was not found. In HP, 46% of the genotypes have “medium”, 28.5% “large” and 25.5% “small” RSAs. In LP, 53.5% have “medium”, 24% “small” and 22.5% “large” RSAs.

## Discussion

### High variability in RSA

The present study reveals a high variability in RSA among genotypes, illustrating the putatively high adaptation capability of the potato crop to root-related stress conditions. Qiao et al. [21] characterized maize RSAs of 174 genotypes and identified potential root traits that can be used as candidate traits conferring stress tolerance. A study examining potato root systems of five tuber-grown cultivars under deficient and sufficient P supply revealed differences among genotypes in root length, root surface area and P-uptake [27]. Another study, analyzing the relation between RSA and drought, demonstrated significant differences among potato genotypes as well [25]. The studies have in common that genotypes with larger root systems tend to perform better under abiotic stress conditions since large-scaled root systems allow plants to exploit a greater soil volume, enabling them to absorb water and nutrients [39, 40]. Similar to the cited papers, we found a significant correlation between total root lengths (NeL) observations of the present study and P-uptake analyzed using the same genotypes in a different experiment (data not shown). Genotypes possessing distinct size-related RSA traits have the potential to serve as raw material for developing water and nutrient efficient potato cultivars.

### P deficiency had a general negative impact on RSA traits

This study showed a strong reduction in nearly all RSA traits under LP conditions. For example, both root length and root surface area of potato genotypes decreased under LP conditions, which indicates an effect of P deficiency on processes related to cell division and growth as described by Balemi and Schenk [41]. However, other studies showed that potato responds to P deficiency by increasing the root surface area via growing additional adventitious roots and root hairs and by maximizing total root length [28, 42]. In our study, however, we did not measure root hairs and the root surface area trait is without root hairs. We saw higher root dry mass as well as a more extended and longer root systems in HP conditions, which agrees with the results of other authors and was also observed in various crops such as peanut, rapeseed and potato [43, 44]. Accordingly, an adequate P supply is important for root system development [27]. However, the extent of P starvation may be causal for contrasting results: While mild to intermediate P deficiency may stimulate root development, severe stress could reduce plant growth to an extent that also root development is affected. A similar effect has been observed in the context of drought stress [24].

Another frequent observation under P deficiency is a strong shift from main to lateral root growth, which leads to a short main root with a large number of long lateral roots [45–47]. These changes result in a shallower root system, which is optimal for topsoil and, thus, P foraging [48]. This, however, does not match with our data. The significant rise of network length distribution (NeLD) shows an increase of roots in the lower parts of the root system. On arable land, P is applied on top of the soil and incorporated into the upper layers by soil tilling. Due to its low mobility in the soil, plants cannot acquire P from deep soil layers, which makes a shallow root system advantageous for P foraging [13, 15]. Since we can assume similar P contents throughout the height of the rhizotrons of the present study, P foraging by root growth towards and in the deeper layers may have improved P acquisition.

Higher root-to-shoot ratios are often reported in the context of P deficiency [49, 50]. Increasing root-to-shoot ratios attribute to a higher transport and utilization of assimilates in the roots as a reaction to P scarcity. However, we did not observe that P shortage changed root-to-shoot ratios significantly. One reason for that could be the short cultivation time in the mini-rhizotrons, which did not lead to observable differences in the root-to-shoot ratio. Garbowski et al. described for several plant species from the families *Asteraceae*, *Poaceae*, and *Plantaginaceae* that root traits can vary significantly with ontogeny [51]. Largest variations were observed for the relative growth rate and the root elongation rate. Other traits, such as specific root length or root diameter, varied only little during seedling development. Trait variation during ontogeny can also differ considerably from species to species [52]. Studies covering this specific topic are still missing for potato and are an interesting objective for future research activities.

### Root-system classification reveals three distinctive rooting types

Apart from general RSA characteristics, three rooting types were identified in the present study: Firstly, a small, thin-rooted system with few lateral roots. The respective potato genotypes are expected to have the least P-uptake availability, since they are able to exploit only a small fraction of the soil. P-uptake data from another experiment support our suggestion. P uptake of the small rooting type was in LP on average 19.5% and 23.7% less than that of the intermediate and large ones, respectively. Secondly, there is an expansive root system, which shows the longest and most extensive network as well as a high number of long lateral roots. Thirdly, there is an intermediate type of RSA, characterized by medium root lengths and spatial extent of the root system. Although it has been shown that an increased RSA resulted in better P-uptake in potato in both the present study and Fernandes et al. [27], da Silva et al. [53] reported that P-efficient wheat genotypes possessed smaller root systems than P-inefficient ones, but they had shallow root angles, which allowed for better topsoil foraging.

Genotypes with both small and large root systems reacted to LP conditions by in- or decreasing their relative root-system size to medium, whereas most accessions with an intermediate root-system size remained in that category. This trend to develop medium-sized RSAs may be one mechanism of adaptation to P limitation. It allows plants to explore a decent soil area at relatively low metabolic costs and acts as a compromise between both metabolic cost and soil exploration. However, some genotypes show relatively stable RSAs under both HP and LP conditions. Examples for that are the cultivar 'Kristall' for the small RSA type and 'Marco' for the large one (Fig. 4). Extreme changes in RSA, e.g., from small to large and from large to small were occurring rarely. Numerous studies indicate that limits of root system plasticity are determined by intrinsic pathways, which are governed by genetic components [48, 54, 55]. This means that potato genotypes have the ability to adapt to suboptimal conditions, but that there are genetically determined limits, which result in relatively small changes in RSA.

### Root systems and crop breeding

Understanding the behavior of root systems to respond to edaphic stresses such as drought or nutrient scarcity is crucial for designing and managing plant breeding programs. It enables the development of cultivars with both improved resource-use efficiency and improved adaptation to abiotic stress. Genotypes within crop species differ in RSA traits and in their ability to take up water and nutrients [19, 41, 56] In this context, RSA traits such as total root length can potentially serve as criteria for developing new cultivars with improved adaptation to adverse environments and with a better resource-use efficiency [57]. Phenotype assessment at very early growth stages can only serve as selection criteria if the measured trait remains stable during development. Total root length (TRL) of the two cultivars 'Cardoso' and 'Kuba' was measured at the end of an experiment with a duration of seven weeks [58]. 'Cardoso' had a significantly higher TRL across different P-nutrition levels, which matches with the results in the HP treatment of the present study. However, 'Kuba' strongly increased TRL in the LP treatment while TRL of 'Cardoso' remained constant. Since TRL of only two cultivars was observed in the previous study [58], it is difficult to draw valid conclusions from the data. Further experiments with a larger number of contrasting phenotypes are required to verify the suitability of RSA traits assessed at very early growth stages as selection criteria in plant breeding.

## Conclusions

Understanding the development and architecture of roots under different P conditions as well as its adaption and plasticity holds great potential for improving nutrient uptake under suboptimal P conditions in the root zone. Our study identified substantial differences in RSA traits across the tested 200 potato genotypes, revealing a high phenotypic variation with potential use in plant breeding. Phenotypic variation in root traits and reactions to P scarcity among diverse potato genotypes can be used to identify quantitative trait loci controlling root architecture. The results of the present study may enable the selection of genotypes serving as germplasm for further research and for breeding P-efficient cultivars.

## Data Availability

All data is stored at the University of Rostock following the guidelines for safeguarding good research practice of the Deutsche Forschungsgemeinschaft (DFG) and available upon request. The plant material is part of the Gross Luesewitz Potato Collections of the IPK and available upon request. The datasets used and analyzed during the current study are available from the corresponding author on reasonable request. Data regarding plant material can be requested from KJD.

## Abbreviations

ANOVA: Analysis of variance
HP: High phosphorus
LP: Low phosphorus
P: Phosphorus
PCA: Principal component analysis
RSA: Root system architecture
TRL: Total root length
